# Using co‐occurrence network topology in assessing ecological stress in benthic macroinvertebrate communities

**DOI:** 10.1002/ece3.5751

**Published:** 2019-10-29

**Authors:** Ariel Levi Simons, Raphael Mazor, Susanna Theroux

**Affiliations:** ^1^ Dornsife College of Letters, Arts and Sciences University of Southern California Los Angeles California; ^2^ Southern California Coastal Water Research Project Costa Mesa California

**Keywords:** co‐occurrence network, ecological index, ecological stress, landscape ecology, stream ecosystems, topology

## Abstract

Ecological monitoring of streams has often focused on assessing the biotic integrity of individual benthic macroinvertebrate (BMI) communities through local measures of diversity, such as taxonomic or functional richness. However, as individual BMI communities are frequently linked by a variety of ecological processes at a regional scale, there is a need to assess biotic integrity of groups of communities at the scale of watersheds. Using 4,619 sampled communities of streambed BMIs, we investigate this question using co‐occurrence networks generated from groups of communities selected within California watersheds under different levels of stress due to upstream land use. Building on a number of arguments in theoretical ecology and network theory, we propose a framework for the assessment of the biotic integrity of watershed‐scale groupings of BMI communities using measures of their co‐occurrence network topology. We found significant correlations between stress, as described by a mean measure of upstream land use within a watershed, and topological measures of co‐occurrence networks such as network size (*r* = −.81, *p* < 10^–4^), connectance (*r* = .31, *p* < 10^–4^), mean co‐occurrence strength (*r* = .25, *p* < 10^–4^), degree heterogeneity (*r* = −.10, *p* < 10^–4^), and modularity (*r* = .11, *p* < 10^–4^). Using these five topological measures, we constructed a linear model of biotic integrity, here a composite of taxonomic and functional diversity known as the California Stream Condition Index, of groups of BMI communities within a watershed. This model can account for 66% of among‐watershed variation in the mean biotic integrity of communities. These observations imply a role for co‐occurrence networks in assessing the current status of biotic integrity for BMI communities, as well as their potential use in assessing other ecological communities.

## Graphical abstract

Using 4,619 sampled communities of streambed benthic macroinvertebrates, found in 67 watersheds covering the state of California, we found patterns of co‐occurrence were associated with the percent of land in a watershed dedicated to human activity. Using five topological measures of the co‐occurrence networks generated in our study, we were able to construct a linear model of watershed health, which could describe 66% of the variation in the health of watershed, as assessed by the California Stream Condition Index. We propose further investigation into the potential for the assessment of the health of ecological communities using topological measures of co‐occurrence networks.

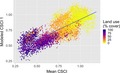

## 1 INTRODUCTION

Humanity can be considered a global scale force for ecosystem engineering (Guerry et al., 2015; Laurance, Sayer, & Cassman, 2014; Vörösmarty et al., 2010). Subsequent to the rise of anthropogenic stressors on the environment, there has been the recognition of the need for ecological monitoring, which can match the scale of human activity (Bergseth, Russ, & Cinner, 2015; Corona, Chirici, McRoberts, Winter, & Barbati, 2011; Foley et al., 2013; Schmeller et al., 2015; Steenweg et al., 2017).

Among the ecosystems being monitored, streams have been of long‐term and ongoing interest. Human activities are both dependent upon their ecological services (Anderson, Glibert, & Burkholder, 2002; Dudgeon et al., 2006) and dependent upon frequently a source of their environmental stress (Carpenter et al., 1998; Paerl et al., 2016). Human‐dominated environments, such as farms, tend to cover large areas. For this reason, there have been efforts to monitor the state of streams across entire watersheds rather than individual streams (Grönroos et al., 2013; Socolar, Gilroy, Kunin, & Edwards, 2016), especially in light of the importance of regional versus local measures of habitat quality with stream communities (Stoll, Breyer, Tonkin, Früh, & Haase, 2016). These biomonitoring efforts have typically focused either on the presence of certain indicator taxa (Fausch, Lyons, Karr, & Angermeier, 1990; Vieira, Séneca, Sérgio, & Ferreira, 2012) or on comparing the composition of communities to an “undisturbed” reference (Kerans & Karr, 1994; Lakew & Moog, 2015; Masese, Raburu, & Muchiri, 2009; Mazor et al., 2015; Silva, Herlihy, Hughes, & Callisto, 2017; Vile & Henning, 2018).

Historically, bioassessments of stream have tended to be based on data sets composed on particular communities, such as BMIs (Cuffney, Brightbill, May, & Waite, 2010; Maxted et al., 2000), organized by morphological classifications. With the advent of high‐throughput metagenomic sequencing, there now exists the potential for rapidly constructing a picture of community composition with greater breadth (Elbrecht, Vamos, Meissner, Aroviita, & Leese, 2017; Stein et al., 2014), taxonomic resolution, and reliability (Baird & Hajibabaei, 2012; Sweeney, Battle, Jackson, & Dapkey, 2011). There is an opportunity then to create a bioassessment framework for BMI communities in which one could readily incorporate community composition data constructed from metagenomic methods (Goodwin et al., 2017; Hering et al., 2018).

Here, we propose the use of co‐occurrence networks to the task of ecological monitoring. These networks represent the likelihoods, represented by edges, of various unique categories of taxa, represented by nodes, co‐occurring in a landscape defined by the spatial extent of the communities sampled and studied. These networks can be constructed from basic ecological data, such as the presence or absence of a set of taxonomic groups across a set of sites (Arita, 2016; Gotelli, 2000; Morueta‐Holme et al., 2016). Co‐occurrence networks have been investigated as a means of inferring ecological patterns, particularly when direct measurement of ecological interactions proves infeasible. For example, the clustering of microbial species into distinct modules within co‐occurrence networks has been used to infer physiochemical niches for various prokaryotic groups (Fuhrman & Steele, 2008; Larsen & Ormerod, 2014; Mandakovic et al., 2018; Ruan et al., 2006; Steele et al., 2011; Widder et al., 2014). In studies of larger organisms, topological measures of these networks have also been used to illustrate a loss in both diversity and the number of significant co‐occurrences between reptiles in response to habitat degradation (Kay et al., 2018). The previous diversity of scenarios where co‐occurrence network topology has been used in ecological analysis then implies that it could also be used to develop a framework for the assessment of the biotic integrity of streams across an entire catchment area (Ahn & Kim, 2017; Moyle & Randall, 1998; Smith & Lamp, 2008).

We hypothesized a number of relationships between ecological stress and five measures of co‐occurrence network topology (Table 1). We chose these measures based on prior analyses of co‐occurrence networks and their relationships with environmental stress, for example, spatial aggregation of species across an environmentally heterogeneous landscape due to variations in their ecological attributes (Bellisario, Cerfolli, & Nascetti, 2010; Borthagaray, Arim, & Marquet, 2014). These hypotheses were then tested using co‐occurrence networks generated from the presence/absence data for benthic macroinvertebrates (BMIs) gathered in streams across the state of California. We used upstream land use as our measure of stress as it was consistently measured at every sample sited, and has been found to be a broad measure of anthropogenic stress in stream communities (Novotny, Bartošová, O'Reilly, & Ehlinger, 2005; Vander Laan, Hawkins, Olson, & Hill, 2013).

**Table 1:** Topological measures of co‐occurrence networks, their ecological relevance, and predicted relationship with an increase in stress due to upstream land use: network size, connectance, mean co‐occurrence strength, modularity, and degree heterogeneity

Topological measure	Ecological relevance	Hypothesized relationship with stress
Network size	The number of unique types of taxa across a set of communities.	(−)
Connectance	The fraction of significant co‐occurrences realized compared to theoretical maximum for a network.	(+)
Mean co‐occurrence strength	Correlation strength between unique types of taxa.	(+)
Modularity	How strongly patterns of co‐occurrence are partitioned into subcommunities.	(+)
Degree heterogeneity	How skewed the distribution of the number of co‐occurrences per unique type of taxa is in a community.	(−)

### 1.1 Ecological stress and co‐occurrence network topology

Prior observations of BMI communities under stress have shown two trends: first, a decline in taxonomic richness (Lenat & Crawford, 1994; Stepenuck, Crunkilton, & Wang, 2002; Voß & Schäfer, 2017); and second, a predominance of members of generalist groups with broad ecological niches (Büchi & Vuilleumier, 2014; Ducatez, Tingley, & Shine, 2014; Mykrä & Heino, 2017). Starting with these trends, we then hypothesized relationships between five topological measures and ecological stress.

#### 1.1.1 Network size

The sizes of our networks were determined by the number of unique BMI genera present within a given set of sampling sites. Ecological stress in BMI communities has been found to be associated with a decline in local taxonomic richness (Ourso & Frenzel, 2003; Paul & Meyer, 2001; Stepenuck et al., 2002; Wallace & Biastoch, 2016). This is especially the case where the stress is due to an increase in upstream land use (Allan, 2004; Sponseller, Benfield, & Valett, 2001; Stepenuck et al., 2002). Given then the correspondence between the taxonomic richness present in a group of sites, and the number of nodes in any resulting co‐occurrence network, we expect network size to be negatively correlated with stress (H1).

#### 1.1.2 Connectance

As the number of edges in a variety of ecological networks may be sensitive to the number of unique taxonomic groups (Dormann, Frund, Bluthgen, & Gruber, 2009; Goldwasser & Roughgarden, 1997; Nielsen & Bascompte, 2007), we then also calculated the connectance (Bell, King, Bohan, & Symondson, 2010). Generalists are expected to have a greater likelihood of co‐occurring with a wider variety of organisms (Fridley, Vandermast, Kuppinger, Manthey, & Peet, 2007), and BMI communities in degraded environments tend to contain relatively more groups classified as generalist (von der Ohe & Goedkoop, 2013; Suga & Tanaka, 2013). We then expect stress to be positively correlated with the fraction of realized versus the potential number of edges (significant co‐occurrences), that is, the connectance, of a resulting co‐occurrence network (H2). With an expected increase in connectance associated with stress, as well as a decline in number of nodes (number of unique BMIs) (Blann, Anderson, Sands, & Vondracek, 2009; Shaver, Maxted, Curtis, & Carter, 1994), we also expect a decline in the number of edges.

#### 1.1.3 Mean co‐occurrence strength

To make additional inferences on shifts in community co‐occurrence patterns in relation to environmental stress, we then determined the mean strength of the co‐occurrences found within each network (Araújo & Rozenfeld, 2014). For this value, we used the mean value of all of the significant correlations, as described by standardized effect‐size scores (Morueta‐Holme et al., 2016), within a network. For any two unique categories of organisms found in a group of communities, the standardized effect‐size score represents the conditional probability, as compared to a null model, of observing one organism given the presence of the other. The mean strength of correlations defining significant co‐occurrences in a network has been observed to decline with the number of edges (Cazelles, Araújo, Mouquet, & Gravel, 2016). We then expect the number of edges in a co‐occurrence network to decline with stress, as described by a mean measure of upstream land use within a watershed, along with a positive correlation between stress and the mean co‐occurrence strength (H3).

#### 1.1.4 Modularity

Prior evidence suggests shifts in communities in response to environmental changes can be better illustrated not just from the number or strength of co‐occurrences, but from their structural arrangement (Fortuna et al., 2010; Thébault & Fontaine, 2010; Tylianakis, Laliberté, Nielsen, & Bascompte, 2010). To measure these structural changes in our co‐occurrence networks, we used the topological measures of modularity, defined here as the proportion of edges that occur within subnetworks less the expected proportion of such edges (Clauset, Newman, & Moore, 2004). With highly modular networks, this would be expected to lead to a co‐occurrence network composed of sparsely interconnected subnetworks.

**Figure 1:**
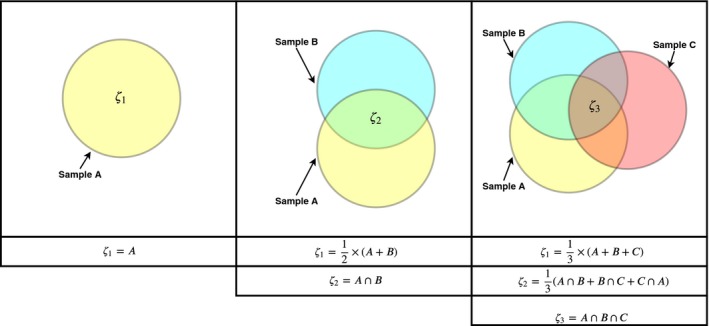
An example of a stressor reducing both the taxonomic richness of three communities, from an initial state (*α*_1_, *α*_2_, *α*_3_) to a degraded state ($\alpha_{1}^{\prime }$,$\alpha_{2}^{\prime }$, $\alpha_{3}^{\prime }$), and the number of unique categories of taxa held in common between communities

Prior observations of stressed watersheds have shown both a decline in local diversity and a rise in landscape diversity as a result of declining taxonomic similarities between individual stream communities (Hawkins, Mykrä, Oksanen, & Vander Laan, 2015; Simons, Mazor, Stein, & Nuzhdin, 2019). Given these prior observations, with regard to changes in patterns of diversity across watersheds in relation to stress, we expect the taxonomic “space” for co‐occurrences to shrink with a rise in stress due to land use (Figure 1), and with it a trend toward the fracturing of assembled co‐occurrence networks into weakly connected subnetworks. Similar relationships, between the modularity of co‐occurrence networks and ecological stress, have also been observed in various ecological communities (Hu et al., 2017; Kay et al., 2018). To assess these trends, we use modularity, the degree to which networks are organized into clusters of weakly interconnected subnetworks (Barberán, Bates, Casamayor, & Fierer, 2012; Clauset et al., 2004). With stress expected to drive greater dissimilarity between communities, we then expect a positive relationship between the modularity of co‐occurrence networks and the levels of stress experienced by the communities from which they are constructed (H4).

#### 1.1.5 Degree heterogeneity

To further investigate changes in the arrangement of co‐occurrences, we determined the degree heterogeneity of each network, a measure of how skewed the distribution of edges per node in a network is toward the most connected nodes (Yan, Martinez, & Liu, 2017). The distribution of edges per node in ecological networks can be indicative of the structure of ecological communities, such as the likelihood of co‐occurrence between generalist and specialist species (Dormann et al., 2009; Williams, 2011).

Prior observations of co‐occurrence networks assembled from communities at increasing levels of anthropogenic disturbance have shown a trend toward the preferential loss of taxa of low degree (Fournier, Mouly, & Gillet, 2016; Tulloch et al., 2016). Additional trends regarding ecological networks have also displayed trends toward the loss of highly keystone taxa due to environmental stresses (Araújo & Rozenfeld, 2014; Morriën et al., 2017). Given both of these trends, the loss of taxa of both high and low degrees, we then expect co‐occurrence networks assembled by communities under stress have a narrow degree distribution and thus a low degree heterogeneity.

Using prior arguments regarding connectance (H2), we also make an additional argument regarding our expected trends in the degree with respect to land use. With ecological networks, connectance has been found to be negatively correlated with the skewness of their degree distributions (Poisot & Gravel, 2014). With degree heterogeneity being a measure of skew for the degree distribution of a network, this then implies that the stress experienced by the communities used to construct co‐occurrence networks will be negatively correlated with their degree heterogeneity (H5).

## 2 MATERIALS AND METHODS

### 2.1 Sample scope

The initial scope of data covered in this analysis consists of 4,984 stream samples from 2,997 unique geographic locations across the state of California, constituting a 23‐year period (1994–2016) (Mazor et al., 2015). Every sample contains the following data: BMIs enumerated and sorted to a standardized level (generally a genus‐level identification except chironomids, which were identified to subfamily; Richards and Rogers, 2006), sample site altitude in meters, U.S. Geological Survey Hydrologic Unit Code 8 level watershed (Seaber, Kapinos, & Knapp, 1987), and the percent developed land use (agricultural, urban, and managed landscapes) within a 5 km clipped buffer of the watershed upstream of the sampling site, and a bioassessment index score (California Stream Condition Index [CSCI]) based on a composite of taxonomic and functional diversity within BMI assemblages (Mazor et al., 2015).

### 2.2 Sample acquisitions and classifications

Approximately 55% of the BMI communities were sampled through a reach‐wide protocol of Peck et al. (2006), with the remainder collected using a targeted riffle protocol, both of which produce comparable data (Gerth & Herlihy, 2006; Herbst & Silldorff, 2006; Rehn, Ode, & Hawkins, 2007). Taxa were identified to one of 334 genera, with each genus then assigned to one of eight functional feeding groups using CAMLnet (Ode, 2003). Of these eight functional feeding groups, we could conclusively assign five of them to either generalist or specialist categories (Barbour et al., 2006; De Castro et al., 2016; Feld & Hering, 2007; Mihuc, 1997; Rawer‐Jost, Böhmer, Blank, & Rahmann, 2000). Using this information, we produced a measure of the number of generalist and specialist genera per sample site.

### 2.3 Calculating the CSCI

Our measure of community biotic integrity at a given stream sample site was done using the CSCI. This index compares observed taxa and metrics to values expected under undisturbed reference conditions based on site‐specific landscape‐scale environmental variables, such as watershed area, geology, and climate (Mazor et al., 2015). This index comprises two sets of measurements using a standardized taxonomy for BMI communities (Richards and Rogers, 2006): the first being a ratio of observed‐to‐expected taxa (O/E), and the second a predictive multimetric index (pMMI) made of six metrics related to ecological structure and function of the BMI assemblage describing the composition of community within a site. The CSCI and its components were designed to have minimal influence from major natural gradients. This in turn has allowed for it to be used as a measure of biological conditions with a consistent meaning in different environmental settings (Reynoldson, Norris, Resh, Day, & Rosenberg, 2006; Hawkins, Olson, & Hill, 2010).

### 2.4 Land use

The type and geographic extent of land use in the upstream vicinity of each sampling site data is derived from the National Land Cover Data set (NLCD) (Homer et al., 2007), with developed land cover measured by the total percent of land cover in a designated area dedicated to agriculture, urbanization, or otherwise managed vegetative landscapes such as golf courses. The designated area for calculating percent developed land cover at each site is defined using a 5 km watershed‐clipped buffer upstream of a stream sampling site using ArcGIS tools (version 10.3; Environmental Systems Research Institute) (Mazor et al., 2015). The values for land use were calculated from NLCD measurements acquired in the year 2000, though it should be noted that the sample sites in our study were located in areas where the percent developed land use was not significantly correlated with time over the duration of this study (*r* = −.02, *p* = .27).

### 2.5 Sample group selection

We first filtered our initial data by selecting watersheds with 15 or more unique samples. This filtering reduced our overall data set from 4,984 to 4,619 unique samples in 2,694 unique geographic locations across 67 watersheds, while containing sample groups with sufficient data density for co‐occurrence network construction. From these remaining samples, we then divided both upstream land use and sample site altitude into quintiles. Samples for network generation were then selected by randomly subsampling 10 samples within each watershed within quintiles of upstream land use and sample site altitude. For each group of 10 samples, we calculated the mean sample site altitude (altitude) and the mean percent developed upstream land use (land use), and for mean geographic separation distance in meters between samples (distance), we used the *distm* function within the R package *geosphere* (Hijmans, Williams, & Vennes, 2012). To obtain a measure of environmental heterogeneity within each sample group, we also calculated the standard deviations on altitude and land use.

### 2.6 Network construction

Co‐occurrence networks were then constructed using the R package *netassoc* (Morueta‐Holme et al., 2016), with the presence/absence site by BMI genera as input. We chose to convert our abundance data to the presence/absence as the most conservative approach with representing our assembled database of BMI communities. Observed co‐occurrences were compared against 100 randomized null communities with the same taxonomic richness as the observed community. The resulting edges were filtered so only correlations representing co‐occurrences, as calculated by standardized effect‐size scores, with a significance and false discovery rate less that 10^–4^ were kept. This process was repeated 100 times, with a set of 8,208 co‐occurrence networks kept for analysis.

### 2.7 Topological measures

Topological measures of our networks, such as size and connectance, were calculated using the packages *igraph* (v.1.2.2) (Csárdi & Nepusz, 2014) and *network* (v.1.13.0.1) (Butts, 2015) in R (v.3.5.1). The mean co‐occurrence strength values were calculated, using the R package *netassoc* (Morueta‐Holme et al., 2016), as the network mean of the significant standardized effect‐size scores. Modularity, defined as the proportion of edges that occur within subnetworks less the expected proportion of such edges, was calculated using the *modularity* function within the *igraph* package (Clauset et al., 2004). Degree heterogeneity was calculated as $\zeta =\frac{<k^{2}>}{<k{>}^{2}}$, where k represents the mean number of edges per node in a network (Yan et al., 2017).

### 2.8 Modeled biotic integrity index

Using the *lm* function in the *stats* R package (v3.5.1, R Core Team, 2018), we constructed a best‐fit linear model to predict the mean CSCI score of a set of samples, our measure of biotic integrity, given the topological measures of their co‐occurrence networks. We then applied a backwards elimination method in order to select topological measures, which make a significant contribution to our model (Pearman, 1997; Snodgrass, 1997). In comparing the AIC scores of linear models after the removal of each topological measure, we found all five were significant. We calculated coefficients for our linear models using a 10‐fold cross‐validation, with 100 repeats, within the “train” function within the R package “caret” (Kuhn, 2008). To determine the relative importance of each topological measure in our linear models, and to adjust for any collinearity between measures as a result, the function *calc.relimp* was used within the *relaimpo* R package (Grömping, 2015). The relative importance of land use, altitude, and distance in describing variations in both the mean CSCI score and our modeled CSCI scores was also done using the *calc.relimp* function.

## 3 RESULTS

In analyzing 8,208 co‐occurrence networks, generated from communities collected from within‐watershed groups with similar values for sample site land use and altitude, we found general support (Table 2) for our hypotheses (Table 1).

**Table 2:** The relative importance of the topological measures used in our modeled stream health indices (*p* < 10^–4^)

Topological measure	*F *(1, 8,208) (Model 1)	Relative importance (%) (Model 1)	*F *(1, 8,208) (Model 2)	Relative importance (%) (Model 2)
Network size	1.4 × 10^4^	44.8	NA	NA
Connectance	704.3	10.0	2,086	19.3
Modularity	151.4	1.3	39.9	1.8
Mean co‐occurrence strength	868.6	7.5	2,462	13.4
Degree heterogeneity	91.6	2.7	387.1	3.3

### 3.1 Trends in co‐occurrence network topology

The size of our co‐occurrence networks declined significantly with a rise in land use (*r* = −.81, *p* < 10^–4^). This reflects a general decline in both the number of genera found in an individual sampling site (*r* = −.52, *p* < 10^–4^) and the number of functional feeding groups (*r* = −.44, *p* < 10^–4^), in relation to land use.

While network size was found to have a strong negative correlation with land use, along with the mean number of edges per node (*r* = −.56, *p* < 10^–4^), we still found that connectance tended to be larger in co‐occurrence networks constructed from groups of stressed communities with a rise in land use (*r* = .31, *p* < 10^–4^). This positive association between stress and connectance appears to reflect a greater relative decline in the number of nodes relative to land use (*r* = −.81, *p* < 10^–4^) than with the number of edges (*r* = −.70, *p* < 10^–4^).

These trends, a rise in connectance despite a decline in network size, may also reflect our observations regarding the relative abundance of unique genera classified by membership of a generalist or specialist functional feeding groups to land use. We found the proportion of genera from specialist functional feeding groups (e.g., shredders and scrapers) tended to decline with land use, while those of generalist functional feeding groups (e.g., gatherers, filterers, and omnivores) tended to increase with land use (Table 3).

**Table 3:** Coefficients of sample site altitude and land use in linear models describing linear models of the percent of genera of BMIs per sample site per functional feeding group (All *p* < 10^–4^ unless otherwise noted)

	Generalist functional feeding groups			Specialist functional feeding groups	
	Gatherers	Filterers	Omnivores	Scrapers	Shredders
Coefficient (land use)	1.6 × 10^–3^	1.5 × 10^–4^	3.5 × 10^–4^	−9.0 × 10^–5^ (*p* < 10^–2^)	−1.5 × 10^–4^
Coefficient (altitude)	8.0 × 10^–6^ (*p* < 10^–2^)	−1.3 × 10^–5^	−5.8 × 10^–6^	−9.0 × 10^–5^	1.1 × 10^–5^

In addition to a rise in connectance, networks assembled from communities with higher land use were on average found to have stronger co‐occurrences (*r* = .25, *p* < 10^–4^). We also found evidence of a negative relationship between both mean co‐occurrence strength and the number of co‐occurrences (*r* = −.32, *p* < 10^–4^), and connectance (*r* = −.24, *p* < 10^–4^). This potentially indicates a preferential loss of weak co‐occurrences in networks assembled from communities under high levels of land use.

Weaker trends were observed with regard to variables, modularity and degree heterogeneity, which describe structural arrangements of co‐occurrences. The mean modularity of our networks (0.35) was found to be both greater than the common modularity threshold of 0.3 (Newman & Girvan, 2004) and greater than that of our randomized null co‐occurrence networks (0.22). Using a Wilcoxon signed‐rank test, we found additional evidence for significant nonrandom structuring in our networks as their modularity values were significantly larger than their randomized null counterparts (*p* < 10^–4^). However, despite evidence of significant network modularity there was only a relatively weak positive correlation with its value and land use (*r* = .11, *p* < 10^–4^).

Across our watersheds, we observe a trend where land use is associated with a slight decline in degree heterogeneity (*r* = −.10, *p* < 10^–4^). However, we did find support for our hypothesis that a decline in degree heterogeneity would be driven, at least in part, by a rise in connectance (*r* = −.60, *p* < 10^–4^). Similar to our results with modularity, we found evidence, using a Wilcoxon signed‐rank test, for significantly higher values for degree heterogeneity in our co‐occurrence networks than their randomized null counterparts (*p* < 10^–4^). The higher mean degree heterogeneity of our co‐occurrence networks (1.82), as compared to that of the null networks (1.14), indicates our networks are skewed more toward a relatively small number of highly connected nodes than what would be expected by chance.

### 3.2 Linear models of watershed biotic integrity using co‐occurrence network topology

Using five measures of co‐occurrence network topology, network size (*N*), connectance (*C*), mean co‐occurrence strength (*S*), modularity (*M*), and degree heterogeneity (*ζ*), a linear model was constructed to best predict the mean value of the CSCI score for a set of samples (Table 2). The relationship between these topological measures and our first modeled mean CSCI score per sample group is as follows:

$$\text{Mean}\;\text{CSCI}=0.3+4.6\times 10^{-3}\times N-1.2\times C-1.8\times 10^{-2}\times S+0.3\times M+0.1\times \zeta$$

**Figure 2:**
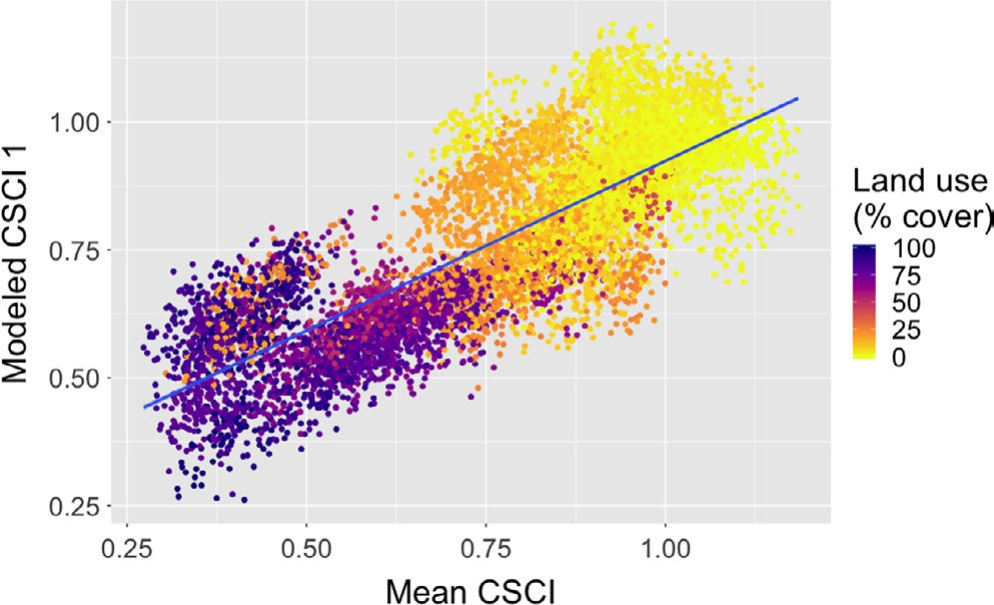
A comparison of the first modeled CSCI and mean CSCI colored by land use (*r* = .81, *p* < 10^–4^). CSCI, California Stream Condition Index

This modeled index of watershed biotic integrity was found to be strongly correlated with the observed variation in the mean value of the CSCI score for a set of samples (Figure 2). After performing a 10‐fold cross‐validation, this model could still account for approximately 66% of the observed variation in the mean CSCI score. This modeled biotic integrity index was also found to vary in accordance with altitude, land use, and distance for a set of samples in a similar fashion as the mean CSCI score, although this first modeled index was less sensitive to altitude and the standard deviation on land use than the mean CSCI score (Table 4). We also observed that most of the variations observed in both the mean and our first modeled CSCI scores were driven by land use and the standard deviation on land use (Table 4).

**Table 4:** The relative importance of altitude, standard deviation on altitude, land use, standard deviation of land use, and distance in describing our linear models of the mean value of the CSCI and modeled index per HUC 8 watershed (All *p* < 10^–4^ unless otherwise noted)

	CSCI	Modeled index 1	Modeled index 2
Proportion of variation due to altitude, *F *(1, 8,208)	690.5	9.0 (*p* < 10^–2^)	189.2
Proportion of variation due to the standard deviation on altitude, *F *(1, 8,208)	26.6	51.8	302.9
Proportion of variation due to land use, *F *(1, 8,208)	2.2 × 10^4^	1.6 × 10^4^	3,082
Proportion of variation due to the standard deviation on land use, *F *(1, 8,208)	776.2	212.5	217.7
Proportion of variation due to distance, *F *(1, 8,208)	28.4	168.4	2.8
Relative importance of altitude (%)	11.6	5.2	7.3
Relative importance of the standard deviation on altitude (%)	7.4	4.1	2.7
Relative importance of land use (%)	33.6	35.2	11.9
Relative importance of the standard deviation on land use (%)	20.7	18.4	9.3
Relative importance of distance (%)	1.0	3.3	0.4
Proportion of variance explained by model (%)	74.2	66.3	31.6

Abbreviation: CSCI, California Stream Condition Index.

**Figure 3:**
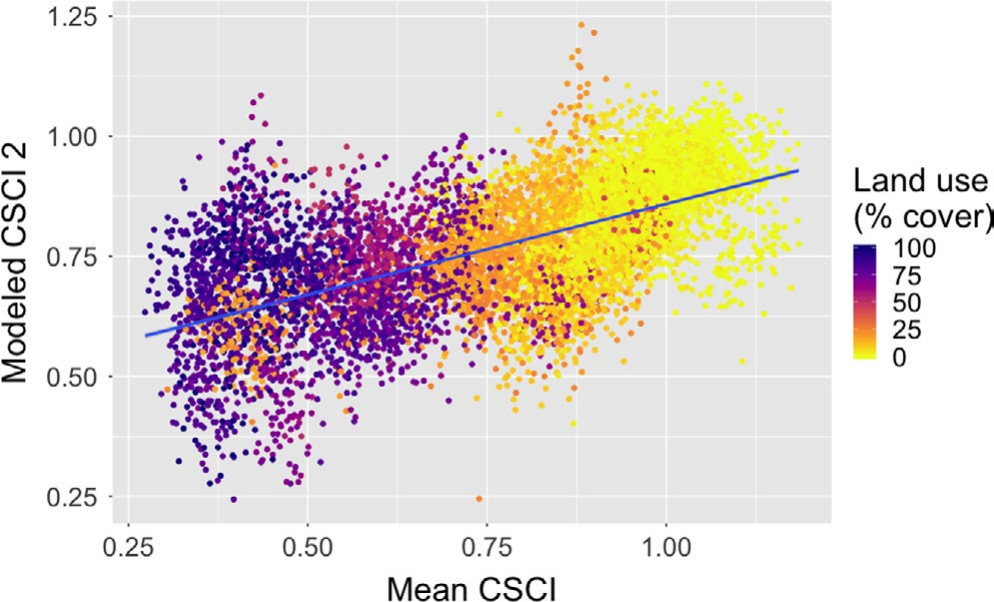
A comparison of the second modeled CSCI and mean CSCI colored by land use (*r* = .61, *p* < 10^–4^). CSCI, California Stream Condition Index

Both network size and the CSCI, our measure of biotic integrity, represent measures based on the taxonomic diversity of sampled communities. To focus on the potential role of the characteristics and configuration of our co‐occurrences, rather than measures of local diversity alone, we then generated a second model of the mean CSCI with network size removed from our list of topological measures (Tables 2 and 4). After performing a 10‐fold cross‐validation, we found this second linear model can account for 38% of the observed variation in the mean CSCI score per group of samples, and it exhibits a similar trend compared to the mean CSCI as with our first model (Figure 3).

## 4 DISCUSSION

We found changes in patterns of co‐occurrence between genera of BMIs can play a role in describing effects of land use on regional measures of biotic integrity. This is reflected in evidence supporting our hypotheses regarding the relationships between land use and the connectance of our co‐occurrence networks (H2), and the mean strength of their co‐occurrences (H3). Evidence supporting our hypothesis regarding a negative correlation between network size and land use (H1) reflects a well‐established link between environmental stress and both the loss of biodiversity and measures of biotic integrity (Freeman & Schorr, 2004; Garie & McIntosh, 1986; Jun et al., 2016, 2011). We find the importance of network size, along with network connectance and co‐occurrence strength, reinforces prior observations on the importance of both regional and local measures of environmental quality in BMI communities (Stoll et al., 2016).

Trends relating the arrangement of co‐occurrences within of our networks, as described by our hypotheses regarding modularity (H4) and degree heterogeneity (H5), were less clear. This may reflect limitations in our use of co‐occurrence rather than co‐abundance networks. However, prior evidence from assessments of biotic integrity for stream communities of BMIs has shown a strong correlation between results generated using community data sets described through the abundance or presence/absence (Beentjes, Speksnijder, Schilthuizen, Schaub, & Hoorn, 2018). The more fundamental issue may stem from differences between networks assembled from co‐occurrences rather than interactions.

Analyses of co‐occurrence networks have been used to identify candidate keystone taxa (Berry & Widder, 2014), potential species interactions (Veech, 2013), and the simplification of communities under ecological stress (Araújo, Rozenfeld, Rahbek, & Marquet, 2011). Though inferring co‐occurrences, rather than verifying interactions, is a far more tractable problem in complex ecological systems, we must acknowledge that co‐occurrences do not necessarily imply interactions. An underlying caveat with analyses involving co‐occurrences is that various types of ecological interactions, such as mutualism or similar environmental requirements, may produce similar patterns of co‐occurrence (Ovaskainen, Hottola, & Siitonen, 2010). In the context of our study, we observed trends between land use and both network connectance and co‐occurrence strength with our BMI communities, which may reflect changes in patterns of interaction between members of generalist genera. However, our co‐occurrence networks may also be incorporating information beyond potential interactions between species, such as the tendency of organisms with similar ecological niches to form co‐occurrences, or for dispersal limitation to tend to limit them (Morueta‐Holme et al., 2016). While co‐occurrence networks, such as the ones we have constructed, may only describe potential interactions, they can still provide useful indications of changes in ecological systems (Freilich, Wieters, Broitman, Marquet, & Navarrete, 2018).

Even with these limitations we found, a simple linear model composed of topological measures of co‐occurrence networks could describe a significant portion of the observed variation in the biotic integrity of our BMI communities (Table 4). Analysis of these models also suggests the topology our networks reflect more than changes in local biodiversity. While network size contributes a sizeable portion of the observed variation in biotic integrity, its removal still leaves more than half of the remaining explanatory power of our linear model of the mean CSCI score (Table 4). This suggests that we are not simply observing a decline in local diversity in response to stress but a change in landscape diversity as well.

Variations in our models appeared to be driven more by both land use and the standard deviation on land use than either altitude or geographic separation distance (Table 4). This reflects our prior observations of this system (Simons et al., 2019), whereby both the mean and standard deviation of land use are strongly correlated with degree of taxonomic dissimilarity between communities. These results are also in agreement with studies of other BMI communities where measures of environmental heterogeneity, such as variations in upstream land use between sample sites (Astorga et al., 2012; Sponseller et al., 2001), appear to drive significant changes in patterns of co‐occurrence (Heino, 2013; Larsen & Ormerod, 2014; Shostell & Williams, 2007; Zhang et al., 2014).

These trends may reflect co‐occurrence patterns unique to stream communities of BMIs. However, the framework we have used to test these hypotheses is not dependent upon the particular identities of taxa present in communities and may have the potential to be applied to other systems. Assessments of the biotic integrity of freshwater ecosystems have been carried out at a variety of spatial scales (Booth et al., 2004; King, Baker, Kazyak, & Weller, 2011; Pratt & Chang, 2012), regions (Jun, Won, Lee, Kong, & Hwang, 2012; Waite et al., 2010; Weigel & Dimick, 2011), and biological communities (Ferreira, Paiva, & Callisto, 2011; Fetscher et al., 2014; Zalack, Smucker, & Vis, 2010). With co‐occurrence networks, we then assert the potential for the development of a more flexible framework for the monitoring of freshwater ecosystems, and find this direction warrants further research.

### 4.1 Synthesis and future directions

It is increasingly becoming feasible to characterize entire ecological communities, from prokaryotes through metazoa, through metagenomic approaches (Baird & Hajibabaei, 2012; Bohmann et al., 2014; Deiner, Fronhofer, Mächler, Walser, & Altermatt, 2016; Jackson et al., 2016). With the ability to generate such broad and deep pictures of multiple communities, there is a commensurate need to create a framework, which could evaluate in general patterns in ecological systems in order to evaluate the biotic integrity of ecosystems. Using these stream communities as an example, our study suggests significant relationships exist between ecological stress and the structure of co‐occurrence networks. We found our co‐occurrence networks reflected changes in the structure of within‐watershed communities in accordance with both the mean and standard deviation on land use and altitude, potentially reflecting segregation in BMI communities with a rise in measures of environmental heterogeneity. Using only a small number of topological measures, we were also able to construct a simple linear model with a close correspondence to a well‐accepted index of biotic integrity. These networks are based on patterns derived from the co‐occurrences of unique organisms, rather than their identities. For such reasons, we believe co‐occurrence networks may have the potential to describe the biotic integrity of a diverse array of ecological communities.

## CONFLICT OF INTEREST

No conflict of interest exists in this manuscript.

## AUTHORS CONTRIBUTIONS

A.L.S. both constructed the co‐occurrence networks used in this study and performed the analyses involved with measures of their topology. R.M. and S.T. were involved in developing the California Stream Condition Index. A.L.S. wrote the first draft of the manuscript, with all authors contributing to subsequent revisions.

## Data Availability

All original data used in this study can be found on the KNB Repository: https://doi.org/10.5063/F10K26VR.
